# Economic-environmental assessment of silvo-pastoral systems in Colombia: An ecosystem service perspective

**DOI:** 10.1016/j.heliyon.2023.e19082

**Published:** 2023-08-11

**Authors:** Danny Fernando Sandoval, Jesús Fernando Florez, Karen Johanna Enciso Valencia, Mauricio Efren Sotelo Cabrera, Burkart Stefan

**Affiliations:** International Center for Tropical Agriculture (CIAT), Tropical Forages Program, km 17 recta Cali-Palmira, Cali, Colombia

**Keywords:** Environmental benefits, Methane emissions, Microclimatic regulation, Heat stress, Sustainability, Ecosystem services, Cattle

## Abstract

Cattle production in Colombia has an important social and economic role but causes considerable environmental impacts, such as deforestation and greenhouse gas emissions by ruminants, particularly methane. Thus, technological innovations aimed at reducing these impacts must focus on both economic and environmental sustainability. Silvo-pastoral systems (SPS) offer productivity increases while generating environmental benefits and ecosystem services and are therefore at the center of debate around sustainable production alternatives. The objective of this article is to evaluate the economic-environmental performance of two proposed SPS for a cattle fattening system for the Colombian context: (i) *Urochloa brizantha* cv. Toledo and (ii) *Urochloa* hybrid cv. Cayman, both in association with *Leucaena leucocephala* trees for browsing and shade provision. They are compared with the respective base scenarios of only using the grasses in monocultures. The study consists of a financial analysis, which estimates potential profitability increases in beef production in the SPS, and an environmental evaluation, which estimates the monetary values of microclimatic regulation and reduction of methane emissions. The value of methane emission reductions is then integrated into a combined economic-environmental evaluation. Results show that both SPS improve the profitability indicators of the production system and reduce the probability of economic loss. Likewise, the reduction of methane emissions in the SPS is estimated at US$6.12 per cattle, and the economic value of microclimatic regulation at US$2,026 per hectare.

## Highlights

• The studied silvo-pastoral systems (SPS) improve profitability of cattle fattening
• The studied SPS help reducing methane emissions, valued at > US$6/beef cattle
• A SPS with 1,000 beef cattle saves annual methane emissions of 32 passenger cars
• The SPS generate shade and reduce heat stress of cattle, valued at > US$2,000/ha
• The SPS are thus a valuable option for mitigation and adaptation in Colombia

## 1 Introduction

Cattle farming is among the most important activities within the agricultural sector in Colombia. According to Fedegan [1], it contributes to about 1.4% of the national, 21.8% of the agricultural, and 48.7% of the livestock gross domestic product of the country. The cattle sector generates employment for more than one million Colombians, mainly in fattening (131,899 jobs), breeding (267,581), dual-purpose cattle farming (530,739), and dairy farming (138,542). About 23 million hectares of land are used for pastures and forage production and the transformation of native landscapes and forests into pastures generates impacts on the different ecosystems of the country. Over the last decades and in the face of growing international concerns on climate change, environmental degradation, and the role cattle farming holds in that regard [2], the sector has been pushed into the center of debate on environmental impacts in Colombia, particularly on those related to deforestation, greenhouse gas (GHG) emissions, and loss of biodiversity [3].

The relationships between cattle farming and the environment are being thoroughly studied by researchers across the globe. In Colombia, several studies have highlighted the strong role cattle farming plays in the reduction of the national forest cover, i.e., in the Amazon, in land grabbing and speculation, and in GHG emissions [[4], [5], [6], [7], [8], [9], [10], [11], [12], [13], [14], [15], [16], [17],^18^,^19^].

To mitigate these negative environmental externalities, focus is put on the development of technical, management, institutional and policy, and market solutions that enhance the sustainability of the sector while maintaining or increasing its social and economic value – a concept defined as sustainable intensification, also aimed at responding to the increasing demand for animal source foods, especially in developing countries [[20], [21], [22], [23], [24], [25], [26], [27], [28], [29], [30], [31], [32], [33], [34], [35], [36], [37], [38], [39], [40]].

Among these solutions, silvo-pastoral systems (SPS) stand out and are considered as a silver bullet solution by policy and decision makers in Latin America, especially in Colombia where a supportive policy framework is being developed [34] and several intervention initiatives are taking place in different regions of the country [[41], [42], [43], [44], [45], [46]]. SPS are combinations of trees, shrubs, and forage grasses, that can be planted in different intensities (e.g., living fences, shade trees, intensive silvo-pastoral systems), aimed at increasing the available quantity and quality of animal feed and thus, animal productivity, while reducing the environmental impacts of traditional, extensive grazing systems [47]. They contribute to increasing soil productivity and quality, carbon accumulation, shade provision and microclimatic regulation (reduced heat stress in cattle), and biodiversity conservation, as well as to reducing soil erosion, among others [[48], [49], [50], [51]]. In short, SPS have a high environmental value and allow for a simultaneous pursuit of productive (milk, beef, timber, fruits) and environmental objectives through the integration of ruminants, grasses, shrubs, and trees [48,50,[52], [53], [54]]. Recent studies on the economic benefits of selected silvo-pastoral system settings in Colombia have shown that their establishment leads to improvements in profitability indicators (such as the Net Present Value, Internal Rate of Return, Benefit-Cost Ratio, or Payback Time) and reduces vulnerability to external factors, such as the climate or input and output price fluctuations [[55], [56], [57]]. The mentioned economic and environmental benefits make SPS an attractive alternative for cattle producers [51] and governments [34] since, when properly managed, they promote sustainability and landscape conservation benefits while contributing to income generation and diversification and food security [47,[58], [59], [60], [61], [62], [63]].

Despite these benefits, the adoption of SPS in Colombia, like in other Latin American countries [64], is still low [39,57,65,66], which coincides with observations made in the adoption process of other planted forages in the country [55,[67], [68], [69]] In addition to technology-specific characteristics and producer preferences, the adoption of cultivated forages and SPS, as for other farming innovations [70,71], is influenced by numerous social, financial, economic, political, and natural factors, such as risk aversion, access to knowledge and extension, labor availability, access to productive inputs, access to credit, land tenure, regulatory frameworks, incentives, and conflict, among others [14,15,34,39,[44], [45], [46],^53^,^65^,^67^,[72], [73], [74], [75], [76], [77], [78], [79], [80], [81], [82], [83], [84], [85]]. When it comes to agroforestry systems, under which SPS can be allocated, adoption becomes an even more complex process since farmers need to make decisions and know about different practices and management strategies, such as on how to select, combine, plant, and manage grasses, shrubs, and trees [54,65,66]. While technical information on cultivated forages and SPS is increasingly being developed and disseminated, often with the help of information and communication technologies [42,[86], [87], [88]], information on the economic benefits of integrating new feeding technologies into cattle systems both from a productive and environmental perspective is mostly being neglected. Land-use and adoption decisions by the cattle farmers, however, are strongly influenced by the profitability promises that these technologies can generate [89], converting profitability indicators thus in important attributes to incentivize or support adoption processes.

Against this background, the present study aims at evaluating the profitability of implementing SPS in Colombia, considering both changes in animal productivity and the provision of environmental benefits and ecosystem services. Particularly, the implementation of two different SPS, namely (i) *Urochloa brizantha* cv. Toledo + *Leucaena leucocephala* trees (SPS Toledo) and (ii) *Urochloa* hybrid cv. Cayman + *Leucaena leucocephala* trees (SPS Cayman) is contrasted with the establishment of two monoculture grass systems commonly present in Colombia: (i) *Urochloa brizantha* cv. Toledo (M Toledo) and (ii) *Urochloa* hybrid cv. Cayman (M Cayman).

For this purpose, a discounted free cash-flow model is applied for each scenario, considering all cost and income flows for a period of 10 years, interest rates, producer price indices, market prices, and animal liveweight gain under the different investment alternatives. In a second step, a Monte Carlo simulation and sensitivity analyses are applied. Regarding the provision of environmental benefits and ecosystem services provided by the SPS, the monetary values of avoided methane (CH_4_) emissions and microclimatic regulation were considered. In this way, the analysis is made up of two main components, namely (i) a financial analysis, which estimates the profitability of beef production in SPS and thus, the financial viability of an investment in SPS, and (ii) an environmental evaluation, which estimates the monetary values of the environmental benefits and ecosystem services obtained by implementing the proposed SPS investments. The monetary value retrieved from the environmental evaluation of CH_4_ emissions reductions is then integrated into an economic-environmental evaluation to provide a comprehensive sustainability analysis. Data was derived from a controlled trial at the campus of the International Center for Tropical Agriculture (CIAT) in Palmira, Colombia, during one year from April 2021 to April 2022.

This document is structured as follows: Section two provides insights into literature on both the economic and environmental evaluation of SPS. Section three describes the sources of information, methodology, and assumptions used in this study. The results of both evaluations and their integration are presented in section four and then contrasted with findings from other studies in the discussion in section five. Section six concludes and provides recommendations.

## 2 Insights into literature on economic and environmental evaluations of SPS

### 2.1 Economic evaluation of SPS

Although the assessment of financial viability of SPS is increasingly gaining importance in scientific literature, research related to the economic profitability and impacts of cattle production systems has been traditionally focused on the evaluation of implementing improved forages, mostly as monocultures [56,57,68,69,80,90,91], or grass-legume associations [55,92]. Likewise, only few recent studies have focused on providing estimates of the economic-environmental benefits of implementing SPS [93,94].

Among the studies that approach SPS from an economic-environmental perspective, Bussoni et al. [93] present a comprehensive analysis that combines the economic benefits of SPS with environmental objectives in Uruguay through three hierarchical models. Model 1 focused on optimizing the combined Net Present Value (NPV) for cattle production (US$302,935) and forestry (eucalyptus timber) (US$556,578). Model 2 focused on prioritizing the cattle NPV (US$317,307) at the expense of a negative balance of carbon dioxide equivalent (CO_2eq._) emissions (−20,160 tons). Model 3 focused on minimizing the CO_2eq._ emissions (+6,788 tons) in a scenario where environmental aims are pursued at the expense of a difference in the cattle NPV of US$-24,609 compared to Model 2. They conclude that both productive and environmental goals can be integrated, and the environmental goal can be achieved if prioritization happens at a higher hierarchical level. For the State of Sinaloa in Mexico, Cuevas-Reyes et al. [94] found that an intensive SPS with *Leucaena leucocephala* and *Cynodon dactylon* is financially viable and that viability significantly increases when the carbon capture potential is considered and monetized (i.e., NPV, IRR, and B/C increase by 15%, 9%, and 2.5%, respectively).

Regarding the financial viability of SPS, Carriazo et al. [95], through choice experiments, studied the effect of technical assistance on the adoption of SPS in Colombia and found that ranchers value SPS with technical assistance for cattle production with US$290 per ha/year while the value for improved forages as a monoculture with technical assistance is significantly lower (US$128 per ha/year). Also for Colombia, Enciso et al. [57] estimated the economic benefits of integrating *Leucaena diversifolia* trees with the grass *Urochloa* hybrid cv. Cayman in a SPS. Compared to the evaluated monoculture with *Urochloa* hybrid cv. Cayman, the SPS showed strongly improved profitability indicators, such as for (i) the NPV, which depending on the scenario was up to four times higher in the SPS, (ii) the Internal Rate of Return (IRR), which improved from 11 to 22%, (iii) the Benefit-Cost ratio (B/C), which was positive in all scenarios, (iv) the payback period, which was reduced from six to four years, and (v) the minimum area required for obtaining two Colombian minimum salaries, which was reduced from 6.5 to 3.8 ha. Likewise, the probability of obtaining economic loss was lower in the SPS. Other profitability analyses conducted for SPS with *Leucaena leucocephala* in Costa Rica [96], the Caribbean region of Colombia [97], and the state of Michoacán in Mexico [98] report significantly higher IRR for the evaluated SPS, oscillating around 33%. In a review of different intensive SPS arrangements in Latin America, mainly with the species *Leucaena leucocephala* and *Tithonia diversifolia*, Chará et al. [99] highlight the positive impacts the establishment of these systems have on edible dry matter production, meat and milk production, the reduction of chemical fertilizer needs and feed concentrates, and thus, farm profitability. Braun et al. [64], in a summary on SPS for South America, conclude that SPS are economically attractive alternatives, which allow for deriving different products (e.g., timber, beef, milk, fruits) at different times, and that the inclusion of trees results in a more secure long-term income while the beef component is more oriented towards short-term incomes. Other authors, such as da Silva Santos and Grzebieluckas [100] for Matto Grosso in Brazil, Quaresma Maneschy et al. [101] for Pará in Brazil, Alonzo [102] for Belize, Boscana et al. [103] for Uruguay, Rade et al. [104] for Ecuador, Ramírez-Martínez and Salas-Razo [105] for Michoacan in Mexico, and Bernardy et al. [106] for Brazil, have also provided insights into the financial viability of different SPS setups in Latin America, evidencing positive results in most cases.

Moving away from Latin America, other studies can be found for Australia, and particularly Queensland, where *Leucaena leucocephala* was identified as the most profitable legume for SPS, capable of doubling per hectare gross margins when integrated with perennial grasses [107], and if adopted at the regional level, could lead to economic benefits of US$ 69 million per year [108]. Francis et al. [109], in a study on the financial performance of SPS in Queensland, Australia, also found that the implementation of SPS is financially attractive, i.e., when timber production is incorporated.

Although an increasing number of studies has evaluated the financial viability of SPS, only two partially quantify some of the ecosystem services offered by them [93,94], disclosing an important research gap around two topics, namely (i) further evaluating the financial viability of SPS in different contexts and (ii) integrating the monetary values of the offered ecosystem services and environmental benefits in comprehensive economic-environmental evaluations. This, however, raises questions on (i) the type of ecosystem services and environmental benefits SPS can offer, and (ii) the availability of technical data on those ecosystem services that allow for including them into the economic evaluation of SPS.

### 2.2 Environmental benefits and ecosystem services generated by SPS

SPS are conceived as multifunctional and dynamic socio-ecological systems, which result from a historical coevolution, i.e., of relationships, feedback, and dependencies, between local communities and their environment. SPS contribute to shaping a diversified landscape structure and configuration that fosters the provision of ecosystem services and environmental benefits to society and the planet [49].

In addition to providing environmental benefits, such as the mitigation of GHG emissions [[110], [111], [112], [113], [114]], SPS provide numerous ecosystem services. Several studies [[48], [49], [50], [51],^115^] have documented evidence on the provision of ecosystem services by SPS, such as microclimatic regulation, carbon storage and sequestration, soil conservation, and the creation of habitats for biodiversity. These environmental advantages of SPS have allowed linking incentive programs such as Payments for Ecosystem Services (PES) that seek to promote the conservation of water sources and ecosystems through sustainable production schemes [61,75,[116], [117], [118], [119], [120], [121]] and become a financial option for producers to partially monetize the environmental value of implementing SPS on their farms.

In Colombia, Vallejo et al. [122] have studied the benefits SPS have on soil quality and nutrient recovery, and found them to be viable alternatives to improve soil quality and metabolic function, which is reflected in the significant increase in microbial biomass, biomarkers, and enzymes. For their part, Polanía-Hincapié et al. [123] carried out a field study that aimed at evaluating changes in the physical quality of the soil that comes along with the subsequent transition from traditional cattle management to SPS in the Colombian Caquetá department. Their results indicate that the implementation of SPS is an efficient strategy to restore the physical quality of the soil in degraded pastures, and contributes to increasing pasture productivity, while indirectly decreasing the pressure of deforestation in the Amazon basin. Martínez et al. [124] have studied the impacts of SPS on soil quality parameters in degraded soils in the Colombian Sinú river valley, showing that SPS helped in increasing soil pH and nutrient availability. Rivera et al. [51], on the other hand, explored the potential benefits that SPS have on the conservation and biodiversity of ecosystems. They conducted a study on the ant fauna in cattle farms in the La Vieja river basin in Colombia, analyzing the relationships between tree cover and the diversity and composition of ant species in different cattle systems. They found that treeless pastures had less than half the number of ant species than pastures that integrated for example *Leucaena leucocephala* trees. Mosquera et al. [125] evaluated the effects of land use changes on soil organic carbon, carbon content, and primary forest stocks, by comparing degraded pastures with improved pasture systems and SPS in the Colombian Amazon. Their results indicate that both the introduction of improved pastures and the implementation of SPS in degraded pastures are feasible alternatives for carbon sequestration. Prior to this, Ibrahim et al. [126], in a study in Colombia, Costa Rica, and Nicaragua, have documented the carbon storage potential of SPS in soil and biomass in rangelands.

## 3 Materials and methods

### 3.1 Description of the evaluated technologies

This study assesses the economic-environmental benefits of implementing two different SPS in Colombia, namely (i) *Urochloa brizantha* cv. Toledo + *Leucaena leucocephala* trees (SPS Toledo) and (ii) *Urochloa* hybrid cv. Cayman + *Leucaena leucocephala* trees (SPS Cayman). These SPS are contrasted with the establishment of two monoculture grass systems commonly present in the country, namely (iii) *Urochloa brizantha* cv. Toledo (M Toledo) and (iv) *Urochloa* hybrid cv. Cayman (M Cayman).

*Leucaena leucocephala* is a shrub legume species that attains a height of 7–18 m as it matures. It possesses a remarkable ability to regenerate vigorously after browsing or substantial pruning. Its branches display a notable degree of flexibility without succumbing to tearing. Additionally, it is highly palatable, and its capacity to fix atmospheric nitrogen is beneficial for the growth of associated grasses. This species thrives across a wide range of elevations, from sea level up to 1,600 m above sea level. It flourishes in areas with annual precipitation ranging between 500 and 3,000 mm, distributed either in a unimodal or bimodal pattern. Furthermore, it can withstand temperatures spanning from 25 to 30 °C. While it exhibits considerable drought tolerance, *Leucaena leucocephala* does not fare well in shaded environments. Adequate sunlight, ranging from 800 to 1,500 h per year, is essential for its optimal growth. The species is adaptable to neutral or alkaline soils and can thrive in stony terrains. However, it does not tolerate waterlogged conditions, acidic soils with high aluminum ion saturation, or intense and prolonged frosts below −4 °C [127,128].

*Urochloa brizantha* cv. Toledo stands as a perennial grass, originating directly from the *Urochloa brizantha* CIAT 26110 accession. It showcases adaptability across a broad range of climates, thriving in sub-humid tropical conditions with dry intervals lasting 5–6 months and an average annual rainfall of 1,600 mm. Similarly, it flourishes in humid tropical environments receiving rainfall exceeding 3,500 mm/year. While it can adjust to acidic soils of limited fertility, its optimal growth occurs in soils of moderate to high fertility. This grass is susceptible to infestations by cercopids and spittlebugs within pastures. Establishing it can be accomplished through either seeding or vegetative propagation. Depending on the chosen method (broadcast or furrow) and seed quality, a sowing density of 3–4 kg/ha is recommended. During rainy periods, this grass can sustain a variable animal stocking rate ranging from 2.5 to 3 Tropical Livestock Units (TLU) per hectare. Its growth potential is also applicable to cut-and-carry systems, as it can reach a height of 1.60 m. Dry matter (DM) production averages fluctuate, measuring 25.2 t/ha in the dry season and 33.2 t/ha during the rainy season. It is feasible to conduct cutting every 8 weeks [129].

*Urochloa* hybrid cv. Cayman emerged as an interspecific hybrid originating from collaborative efforts between the International Center for Tropical Agriculture (CIAT) (breeding), the Centro de Investigación de Pastos Tropicales (CIPAT) (evaluation and selection), Grupo Papalotla (introduction, commercialization). This grass variety exhibits a remarkable ability to thrive in moist environments and displays a notable adaptability to soils predisposed to waterlogging. Its growth pattern is characterized by a decumbent form, generating an abundance of runners. Particularly noteworthy is its responsiveness to humidity, which triggers a transformation in growth behavior. In its early stages, Cayman develops decumbent stems, fostering the emergence of shoots and roots at the nodes. This unique adaptation enhances nutrient absorption and oxygen production, particularly beneficial in poorly drained conditions. Beyond its waterlogging resistance, this hybrid boasts drought tolerance, marked palatability, stoloniferous growth, and an innate resilience against pests and diseases. Moreover, it facilitates the sustenance of higher animal stocking rates, making it an asset for cattle management [130].

### 3.2 Data sources and description of the treatments

The data used for the financial analysis were obtained from a field trial established at the CIAT campus in Palmira, Colombia. According to Holdridge [131] the study area can be classified as pre-montane wet forest (bh-P), which is located at an elevation of approximately 1,000 m.a.s.l. The annual precipitation is 1,045 mm and the rainfall regime is bimodal (March to April, October to November). The average temperature is 23.8 °C and the relative humidity 75%. The trial was established on a fertile, clayey Mollisol (40–60% clay content) [132] with a pH of 7.54, CEC of 16.4 cmol/kg, good drainage, and P, Ca, Mg, and K concentrations of 25 ppm, 7.87, 6.17, and 0.82 cmol/kg, respectively.

Four treatments were established, namely (i) SPS Toledo, (ii) SPS Cayman, (iii) M Toledo, and (iv) M Cayman. Measurements were made for one year, between April 20, 2021 and April 20, 2022, to cover all four seasons occurring at the location (two rainy and two dry seasons). The overall aim of the trial was the evaluation of animal liveweight gains in two different scenarios, SPS and grass monocultures. Each of the four treatments was carried out in an area of 1 ha, which were divided into 3 plots (0.33 ha each) for rotational grazing. In the two SPS treatments, the proportion between the respective grass and the *Leucaena leucocephala* trees was 70/30, and the tree density 2,000 trees per ha (25% for shade, 75% for browsing).

A total of 14 Brangus cattle were utilized for grazing, evenly distributed across the two scenarios (SPS and M) in a 50% ratio. The average age upon entry of the animals was 18.6 months, with a mean of 19.1 months for the M treatment and 18.1 months for the SPS treatment. At the commencement of the trial, the typical weight of the animals in the M treatment stood at 349 kg, while in the SPS treatment, it was 345 kg. The grazing and rest intervals were maintained at 12 and 45 days, respectively. In terms of stocking rate, the M treatment accommodated 3 animals per hectare, whereas the SPS treatment hosted 4 animals per hectare.

Regarding the environmental evaluation, following a comprehensive review of available information and data, one notable environmental benefit and a single ecosystem service were considered, namely (i) the mitigation of CH_4_ emissions and (ii) microclimatic regulation. For the analysis of CH_4_ emissions reductions, the study by Gaviria-Uribe et al. [133] was considered. Their research provided estimates of CH_4_ emissions within this experiment across various diets in both M and SPS scenarios. As for microclimatic regulation, an assessment was conducted by measuring the extent of shaded areas within the SPS treatments on September 1, 2022, utilizing the Fields Area Measure application.

### 3.3 Financial analysis

#### 3.3.1 Discounted free cash flow model

In this study, a discounted cash flow model was employed to estimate profitability indicators, facilitating a comparative analysis of various investment options (SPS, M). The assessed indicators encompass NPV, IRR, and B/C, following the guiding principles outlined by Park [134]. These indicators are derived by adopting the most probable values within distinct specified models, aligning with the benefits and costs associated with each investment alternative. The assessment is constructed through a juxtaposition of the calculated profitability indicators for the different treatment scenarios. Costs incurred were categorized into four main areas: (i) establishment and maintenance costs, (ii) opportunity costs, (iii) operational expenses linked to animal health and supplementation, and (iv) labor costs. Conversely, benefits stem from the cattle's commercialization, with their profitability parameters contingent upon the liveweight gain observed in each of the evaluated treatments.

#### 3.3.2 Model assumptions

For the elaboration of the cash flows, the following technical and economic assumptions were made:

*Technical Assumptions:* All cattle engaged in the treatments were exclusively of the Brangus breed. To formulate the cash flows, stocking rates of 3.0 and 4.0 animals per hectare were adopted for the M and SPS treatments, respectively. These rates remained consistent throughout the analysis period. Liveweight gain was meticulously assessed for all treatments at both the commencement and conclusion of the experiment (Table 1). The feeding duration for the animals was standardized at 365 days across all treatments.

**Table 1:** Summary of the costs and benefits of the different treatments.

Indicator	M Toledo	M Cayman	SPS Toledo	SPS Cayman
Stocking rate (#cattle/ha)	3	3	4	4
Liveweight gain (g/d/animal)	159.25	159.25	239.81	239.81
Animal productivity (kg/ha/y)	723	723	1078	1078
Income from sale of meat (US$/ha/y)	1,501.84	1,501.84	2,007.33	2,007.33
Pasture establishment cost (US$)	468.69	647.04	482.72	814.13
Pasture maintenance cost (US$)	70.93	87.68	52.61	68.39
Pasture renovation cost (US$)	76.94	106.96	79.66	103.56
Maintenance cost *Leucaena leucocephala* (US$)	–	–	15.03	15.03

*Evaluation horizon:* The evaluation horizon was determined based on the typical lifespan of the grass technologies, which is commonly recognized as 10 years [135], spanning from 2020 to 2029. It is worth noting, however, that both evaluated grass varieties can potentially remain productive for a considerably longer duration with proper management practices such as grazing and fertilization. Additionally, the assessed *Leucaena leucocephala* trees generally exhibit lifespans extending beyond 10 years. It is also important to acknowledge that in practical scenarios, farmers frequently exhibit reluctance to renew pastures, resulting in the continued utilization of established technologies for over 10 years. Nonetheless, this often leads to a decline in pasture productivity due to the scarcity of adequate pasture management practices. Nevertheless, to promote the effective adoption and utilization of such technologies, the present article adopts a 10-year timeframe as the technology lifespan.

*Discount rates:* The determination of financing costs was based on the established credit lines for cattle production systems, including those incorporating silvo-pastoral settings, as defined by Finagro, the Colombian Fund for Financing the Agricultural Sector [136]. This discount rate is commonly employed as the representative opportunity cost for capital investments and is closely tied to the risk factors inherent in agricultural endeavors. To encompass the potential impact of variations in discount rates on system profitability, multiple scenarios were explored using DTF (fixed-term deposit rate) + 3%, 5%, and 8%, respectively. The projections for the discount rate were derived from two primary sources: (i) the Bancolombia Annual Report of Economic Projections for Colombia in 2020 [137], and (ii) the macroeconomic projections report for 2020 from the Banco de la República of Colombia, which collates insights from both local and foreign analysts [138].

*Permanent labor:* Following the labor weighting factors outlined by Fedegan [139], a cattle fattening-oriented system requires approximately 2.3 permanent positions for every 100 animals. The salary estimate is calculated based on the prevailing monthly legal minimum wage (MW) for the year 2019, encompassing transportation assistance, social security contributions, and associated benefits, totaling around US$400 per month. To forecast the evolution of the minimum wage throughout the analysis period, it was assumed that the variation would mirror the anticipated inflation rate for each year, coupled with a labor productivity adjustment set at 1%, in accordance with historical data from national statistics [140]. The labor remuneration used in this analysis aligns with Colombia's salary dynamics, which are anchored by the MW. As reported by DANE [141], approximately 32.2% of the employed population in Colombia received a salary near 0.9 MW in 2020, while 17.8% fell within the range of 0.9–1.1 MW. This nuanced distribution provides insight into how rural area wages correspond to national average wage trends. Considering its proximity to the national average, with minor regional variations, the adopted MW serves as a suitable proxy.

*Inflation:* Incorporating the impact of inflation, the analysis encompassed the estimation of revenues and costs over the evaluation period. Projections for income were based on the Consumer Price Index (IPC) data published by the Banco de la República of Colombia [142]. In parallel, the Producer Price Index (IPP), as calculated by the National Administrative Statistics Department of Colombia (DANE) [143], was employed to gauge production costs.

*Cattle price:* In the context of local weather events and the global impact of the COVID-19 pandemic, the Colombian cattle sector experienced inflationary and external influences, leading to a notable rise in cattle prices [1,42]. Given the exceptional nature of these price fluctuations, the study referred to prices from 2019 for the purposes of analysis.

#### 3.3.3 Quantitative risk analysis

Risk is defined as the potential that the actual investment return will fall short of the expected investment return [134]. Consequently, profitability is intertwined with the volatility of income and cost streams, hinging on the unpredictability of key variables within the investment project, such as yields and market prices. Rural investment endeavors are particularly exposed to unique risks, their outcomes being contingent upon a broad array of variables, many of which lie beyond the investor/producer's control, such as climatic factors. In this context, it becomes imperative to integrate risk considerations into the assessment of profitability indicators for each evaluated investment. To this end, a Monte Carlo simulation model was employed. Monte Carlo simulation involves generating a sample of outcomes based on specified probability distributions [134]. This approach empowers decision-makers to explore potential outcomes and gauge the influence of risk on investment project profitability indicators. Executing the simulation necessitates the identification of random input variables, those capable of adopting multiple feasible values, and defining the plausible range for each. Probability distributions are then assigned to these variables, followed by the computation of projected indicator profitability. In the present study, the Monte Carlo simulation was executed using the @Risk software package (Palisade Corporation), involving 5,000 iterations and a confidence level of 95% across all treatments.

#### 3.3.4 Decision criteria

The decision criteria are rooted in the average values of the profitability indicators NPV and IRR, alongside the Benefit-Cost Ratio (B/C), which stem from the simulations conducted for both the M and SPS treatments.

*Net Present Value:* This indicator represents the present value of the cash flow stream, calculated as the aggregate of discounted benefits subtracted from discounted costs [144].

*Internal Rate of Return:* As outlined by Gittinger [144], an alternative approach to assess the value of a project using the revised cash flow involves identifying the discount rate that renders the net value of the cash flow equivalent to zero. This discount rate is referred to as the Internal Rate of Return (IRR), which, in essence, signifies the average return on the investment expenditure throughout the entirety of the project's lifespan. The equations for the NPV (Equation (1)) and IRR (Equation (2)) are as follows:

(1) $${NPV}_{\left(Mean\right)}=\sum_{t=0}^{n}\frac{E\left(FC_{t}\right)}{{\left(1+r\right)}^{t}}$$

(2) $$IRR_{\left(Mean\right)}=\sum_{t=0}^{n}\frac{E\left(FC_{t}\right)}{{\left(1+r*\right)}^{t}}=0$$

where *E(FC*_*t*_*)* is the expected value of the net profit flow for period *t*, Var*(FC*_*t*_*)* is the net profit flow variance for period *t*, *r* the real discount rate, r* the internal rate of return, and *t* the evaluation horizon of the project.

*Cost-benefit analysis:* Cost-benefit analysis (CBA) is a structured economic evaluation tool that involves a comparison between the costs and benefits of a given project. Functioning as a comprehensive analytical framework, CBA serves as a means to assess and appraise both public and private ventures [145]. Economically speaking, the Benefit-Cost Ratio (B/C) applies a discount mechanism to both the influx of benefits and the outflow of costs, utilizing a rate that approximates the opportunity cost of capital. This approach establishes a quantifiable linkage between the discounted value of project benefits and the corresponding value of the discounted costs associated with it. The equation for the B/C (Equation (3)) is as follows:

(3) BC=∑Bt/(1+r)t∑Ct/(1+r)t

where *Bn* are the benefits in period *t*, *Cn* the costs in period *t*, *r* the real discount rate, and *t* the evaluation horizon of the project.

The Benefit-Cost Ratio (B/C) provides a rough indication of the effectiveness of funds allocated to an investment, offering a transparent gauge of the viability and allure of an investment project. The decision criteria can be summarized as follows:

• B/C > 1: This suggests that the revenue generated by the investment will surpass all associated expenditures, indicating project profitability.
• B/C = 1: This signifies that both the generated revenue and expenses of the investment will be equivalent. The decision to proceed with the investment remains neutral.
• B/C < 1: This implies that the generated revenue from the investment will fall short of covering all incurred expenses, indicating project unprofitability.

### 3.4 Environmental evaluation

The environmental evaluation encompasses the economic appraisal of environmental advantages and ecosystem services arising from the SPS as opposed to the M treatments. In terms of environmental benefits, the replacement of traditional M systems with SPS leads to a reduction in CH_4_ emissions. In the realm of ecosystem services, three prominent aspects have been identified: (i) microclimatic regulation achieved through the shading effect of trees, (ii) carbon storage and sequestration within tree biomass, and (iii) soil nitrogen fixation. However, for the scope of this study, the focus is on valuing the environmental benefit of CH_4_ emission reduction and the ecosystem service of microclimatic regulation. This decision is due to the absence of available technical ecological measurements for the assessment of carbon storage and capture, as well as soil nitrogen fixation.

To assess the value of CH_4_ emission reduction, the market price method [146] was implemented, leveraging data from major global Tradable Emission Permit Systems and economic mechanisms like CO_2_ taxes. The market price method establishes the economic worth of CO_2eq._ (*v*) by multiplying the volume of avoided CO_2eq._ emissions (*q*) with the average market price for emissions and/or CO_2_ taxes as sourced for the analysis year (*p*) (as represented in Equation (4)). In order to ascertain *q*, this study draws upon findings from Gaviria-Uribe et al. [133], who indicated that for every gram of animal liveweight gain, emissions of 0.36 and 0.33 g of CH_4_ occur in the M Toledo and SPS Cayman treatments, respectively.

(4) v=q*p

To appraise the reduction in CH_4_ emissions, certain assumptions were made regarding the initial (220 kg) and slaughter weight (450 kg) of the cattle. These assumptions enabled the utilization of daily liveweight gain data from different treatments and the CH_4_ emissions factor per gram of liveweight gain. This information was employed to estimate emissions generated up until the point of the animal's sale. Since Gaviria-Uribe et al. [133] exclusively provide data for M Toledo and SPS Cayman, it was deduced that CH_4_ emissions for SPS Toledo would be akin to those of SPS Cayman. Consequently, identical values as those for SPS Cayman were employed for assessing the monetary worth of CH_4_ emissions reduction in SPS Toledo.

To assess the monetary value of the microclimatic regulation ecosystem service, the method of avoided costs [146] was employed. This approach involves estimating the expenses associated with the establishment and upkeeping of a gray infrastructure that mirrors the shade coverage provided by the trees within the SPS. The chosen gray infrastructure for comparison is a shade mesh, as it is the prevalent choice for cattle systems in Colombia. In this context, the monetary value of the microclimatic regulation ecosystem service (*v*) is determined by multiplying the area encompassed by the shade (*q*) with the average costs associated with installing and maintaining the selected gray infrastructure (*c*), as illustrated in Equation (5).

(5) v=q*c

### 3.5 Limitations of this study

It is essential to acknowledge several limitations that should be taken into account when interpreting the findings presented in this financial analysis.

Firstly, due to the age of the cattle upon entering the SPS treatments, it was not feasible to measure initial-stage liveweight gain, a crucial component of the productivity curves associated with grazing. While the outcomes for the SPS treatments were more favorable compared to the M treatments, the exclusion of initial-stage liveweight gain measurements could potentially impact the results of the SPS treatments.

Secondly, both input and labor costs used in modeling the cash flows for cost, benefit, and other indicator measurements were influenced by external factors, such as the COVID-19 pandemic [42,147]. The pandemic led to disruptions in agro-industrial input supply chains and local price inflation, directly affecting the implementation costs of the studied treatments. Consequently, the results of this study are significantly shaped by the global and local macroeconomic context that unfolded since 2020. This economic environment also impacted on the projections incorporated into the cash flow analysis. Considering the economic uncertainty at both national and international levels, inflation, wage, and producer price index forecasts used to project benefits and costs demonstrated higher-than-expected increases. Despite consulting multiple sources to compare forecasts from both public and private institutions, the prevailing trend was towards upward adjustments due to the economic uncertainty of 2020. To mitigate these extraordinary effects in the analysis, 2019 prices were adopted as the foundation for this study.

Furthermore, it is important to recognize that this analysis was conducted on an experimental research scale within CIAT's campus. Consequently, the estimated productivity and costs may deviate from scenarios on farmers' fields under commercial conditions.

In the realm of environmental evaluation, it is important to highlight that SPS generate various other environmental benefits and ecosystem services that were not quantified in this study. These include carbon storage and sequestration in tree biomass, soil nitrogen fixation, fertilization of soil through animal excrement, and the creation of habitats for various species. These services were not valued due to the lack of ecological estimates of physical units for each service specific to the SPS setups of interest. Furthermore, a social valuation exercise was not undertaken as it would have necessitated an additional experimental design involving participation from producers to ascertain values based on their perceptions. Additionally, the avoided cost method used reference prices from the Colombian Cauca Valley department, where the trial was located, which may yield different outcomes if applied to other regions within Colombia.

## 4 Results

### 4.1 Financial analysis

One of the most important basic aspects for the financial analysis of SPS and M is the cost structure associated with the setup and management of the technologies, given that these largely define the investment decision producers need to make when adopting and implementing a new feed production system. As can be observed in Table 1, higher costs occur for the establishment of SPS, particularly they increase by 26% and 3% for SPS Cayman and SPS Toledo, respectively, when compared with the M alternatives. Regarding the maintenance and renovation costs, they tend to be slightly lower for the SPS, since less area is sown with the more labor and fertilizer intensive grasses which also need partial renovation after around five years to maintain pasture productivity stable. Regarding the benefits, due to the increased amount and quality of available feed, the animal stocking rates are 33% higher in SPS than in M, which translates into higher daily liveweight gains (+51%), annual per hectare animal productivity (+49%), and annual income from the sale of meat (+34%) in SPS.

Table 2 presents the results for the financial indicators for the M and SPS treatments. A negative NPV was obtained for the two M treatments, precisely of US$-268 and US$-527 for M Toledo and M Cayman, respectively. Both treatments also show a negative IRR, although for M Cayman it is close to zero, and a high risk of obtaining economic loss, 67% and 81% for M Toledo and M Cayman, respectively. The two SPS treatments, on the other hand, show disparate results. As for the NPV, SPS Toledo presents a positive value of US$35, whereas it is negative for SPS Cayman (US$-218), which is related to the significant difference in establishment (+69%), maintenance (+30%), and renovation (+30) costs of the pasture in SPS Cayman compared to SPS Toledo. Likewise, the IRR is positive for SPS Toledo but negative for SPS Cayman, and the risk of obtaining economic loss is high for both. The B/C ratio is slightly positive for the SPS treatments. Despite these rather discouraging numbers, the data show that significant improvements occur in all indicators when implementing SPS instead of M.

**Table 2:** Results for the financial indicators for the different treatments.

Indicator	M Toledo	M Cayman	SPS Toledo	SPS Cayman
Mean NPV (US$)	−268.05	−527.96	35.10	−218.49
Mean IRR (%)	−4.39	−0.06	0.58	−2.39
Risk of economic loss (NPV<0) (%)	67.5	80.8	48.7	59.0
B/C Ratio	1.00	0.99	1.03	1.02

**Fig. 1:**
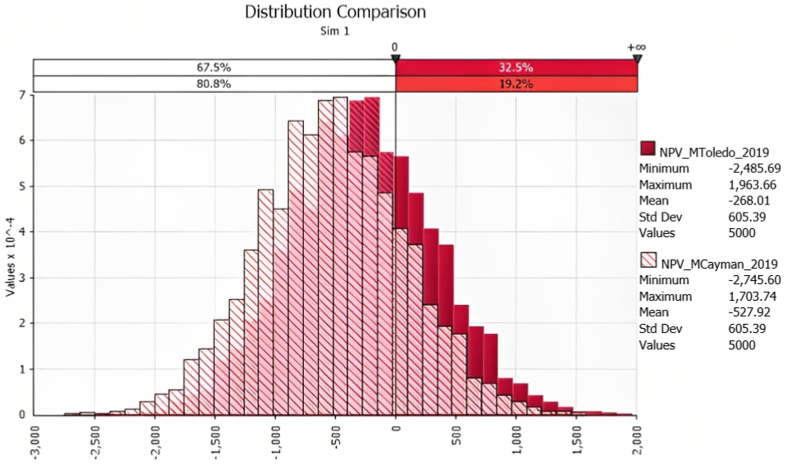
Probability density distributions for NPV for the M treatments (dark red: M Toledo; light red: M Cayman).

**Fig. 2:**
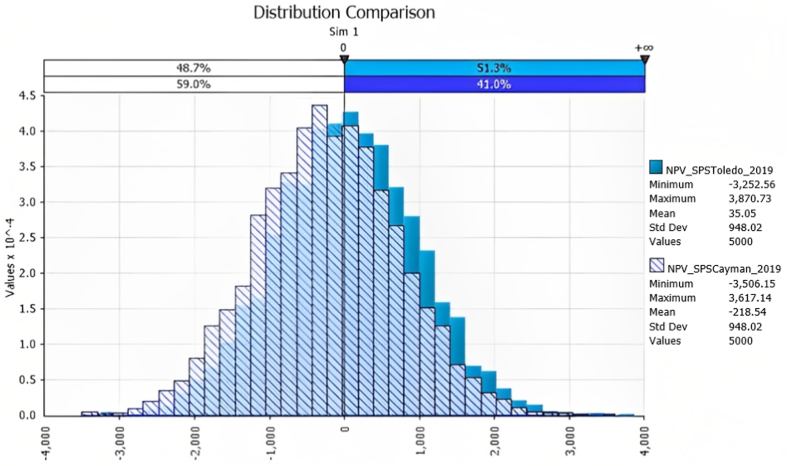
Probability density distributions for NPV for the SPS treatments (dark blue: SPS Toledo; light blue: SPS Cayman).

Fig. 1, Fig. 2 show the results of the Monte Carlo simulation risk analysis for the M and SPS treatments. It can be observed that despite the still high risk of obtaining economic loss (NPV<0) in the SPS treatments (in 49% and 59% of the scenarios generated for SPS Toledo and SPS Cayman, respectively), it is significantly lower than in the M treatments (67.5% and 80.8% for M Toledo and M Cayman, respectively).

### 4.2 Environmental evaluation

#### 4.2.1 Reduction in CH_4_ emissions

Based on the results of Gaviria-Uribe et al. [133] it is found that the SPS Cayman treatment achieves a CH_4_ emissions reduction of 0.03 g per gram of animal liveweight gain, which is equivalent to a reduction of 0.63 g of CO_2eq._ compared to the M Toledo treatment. If this diet was replicated in a larger SPS, for example with 1,000 cattle, a reduction of 145 tons CO_2eq._ could be achieved. Table 3 presents an overview of the data presented by Gaviria-Uribe et al. [133]. The economic value of the reduction of avoided CH_4_ emissions corresponds to a potential income that a producer can access by implementing good environmental practices. This reduction contributes to the objective of climate neutrality in livestock farming, which is encouraged in Colombia by a program for payments for environmental services (PES). The PES program has a special emphasis on supporting livestock producers who implement silvo-pastoral systems and demonstrate environmental benefits such as the CH_4_ emissions reduction presented in this article [119,148].

**Table 3:** Estimated reduction of CH_4_ emissions in the SPS Cayman treatment.

Indicator	M Toledo	SPS Cayman
CH_4_ emissions (g/g liveweight gain)	0.36	0.33
CO_2eq._/CH_4_ equivalence IPCC	21	21
CO_2eq._ (g/g liveweight gain)	7.56	6.93
Liveweight gain (g/d/animal)	273	742
Daily emissions (CO_2eq._ g/d)	2,064	5,142
Initial weight (kg/animal)	220	220
Slaughtering weight (kg/animal)	450	450
CO_2eq._ emissions generated until slaughtering weight is achieved (t)	1.739	1.594
CO_2eq._ emissions generated in a system with 1,000 cattle (t)	1,739	1,594

When consulting the most important global carbon markets [149], the carbon tax in Colombia [150], the minimum price recommendation for carbon credits of the International Monetary Fund (IMF) [151], and the general equilibrium model with the Tradable Emission Permit System in Colombia of the National Planning Department (DNP) [152], an average price per ton of CO_2eq._ of US$45.25 per ton can be defined for the period between January and August 2022 (Table 4).

**Table 4:** Estimation of the market price of CO_2eq._ in 2022.

Market	Price (US$/t CO_2eq._)
Colombian carbon tax 2022	4.72
Price as of the general equilibrium model with the Tradable Emission Permit System in Colombia of DNP	41.00
Minimum price suggested by IMF in 2021	25.00
EU Emissions Trading System (ETS)	83.24
California ETS	30.00
Québec ETS	38.38
New Zealand ETS	76.35
Regional Greenhouse Gas Initiative (RGGI)	15.10
United Kingdom ETS	76.28
Germany ETS	32.41
Average price for 2022	42.25

According to this average price, the monetary value for the environmental benefit of CH_4_ emissions reduction in SPS Cayman can be estimated at US$6.12 per cattle. Considering the stocking rate of the experimental setup of four cattle per hectare, a total monetary value of US$24.49 per hectare can be observed, which is equivalent to an annual value of US$28.83. A replication of SPS Cayman at larger scales, for example in a cattle fattening system with 1,000 animals, could thus generate a monetary value for CH_4_ emissions reductions of US$6,122 (Table 5).

**Table 5:** Estimated monetary value of reducing CH_4_ emissions in the SPS Cayman treatment.

Indicator	M Toledo	SPS Cayman
CO_2eq._ emissions generated in a system with 1,000 cattle (t)	1,739	1,594
Price (US$/t of CO_2eq._)	42.25	42.25
Total cost for CO_2eq._ emissions generated in a system with 1,000 cattle (US$)	73,460	67,338
Economic value of CO_2eq._ emissions reductions in a system with 1,000 cattle (US$)	0	6,122
Economic value of CO_2eq._ emissions reductions (US$/cattle)	0	6.12
Stocking rate (cattle/ha)	3	4
Total economic value of CO_2eq._ emissions reductions (US$/ha)	0	24.49
Total economic value of CO_2eq._ emissions reductions (US$/y)	0	28.83

#### 4.2.2 Microclimatic regulation

Concerning the microclimatic regulation ecosystem service, the SPS treatments encompass an area of 12,082 m^2^ under shade, as determined through field measurements, translating to a shade coverage of 60.4%. Engaging with ten shadow mesh providers in the Cauca Valley Department, the average prices for shadow mesh (per m^2^), poles (per pole), and labor (per day) were ascertained to be US$0.78, US$5.49, and US$9.24 respectively. The anticipated lifespan of the infrastructure is three years.

Thus, should the tree shade coverage within the SPS treatments be replaced by shadow mesh, the total cost over three years would amount to US$12,158, equivalent to US$4,053 annually and US$2,026 per hectare per year. When extrapolated to a larger scale, such as a 1,000-ha cattle fattening system, the monetary value of microclimatic regulation could potentially reach US$2,016,414 per year (as indicated in Table 6).

**Table 6:** Estimated monetary value of microclimatic regulation in the SPS treatments.

Item	Unit	Cost (US$/unit)	Quantity (per unit)	Total cost (US$/3y)	Annual cost (US$)	Annual cost (US$/ha)	Annual value for 1,000 ha (US$)
Shadow mesh	m^2^	0.78	12,082	9,471	3,157	1,578	1,578,425
Poles	Pole	5.49	483	2,651	884	442	441,830
Labor	Day	9.24	4	37	12	6	6,159
			**Total**	**12,158**	**4,053**	**2,026**	**2,026,414**

### 4.3 Integrated economic-environmental evaluation

In the last step of analysis, the monetary value of the environmental benefit of CH_4_ emissions reduction provided by the SPS was integrated into the financial analysis. Table 7 shows how the financial indicators change under this scenario compared to the Base Scenario presented in Table 2.

**Table 7:** Results for the financial indicators for the different treatments and different scenarios.

Scenario	Indicator	M Toledo	M Cayman	SPS Toledo	SPS Cayman
Base Scenario: Economic benefit of the cattle system without considering environmental benefits and ecosystem services	Mean NPV (US$)	−268.05	−527.96	35.10	−218.49
	Mean IRR (%)	−4.39	−0.06	0.58	−2.39
	Risk of economic loss (NPV<0) (%)	67.16	80.95	48.84	59.56
	B/C Ratio	1.00	0.99	1.03	1.02
Scenario 1: Economic benefit of the cattle system + CH_4_ reduction	Mean NPV (US$)	–	–	259.97	6.38
	Mean IRR (%)	–	–	3.00	−0.23
	Risk of economic loss (NPV<0) (%)	–	–	39.2	50.2
	B/C Ratio	–	–	1.04	1.03

**Fig. 3:**
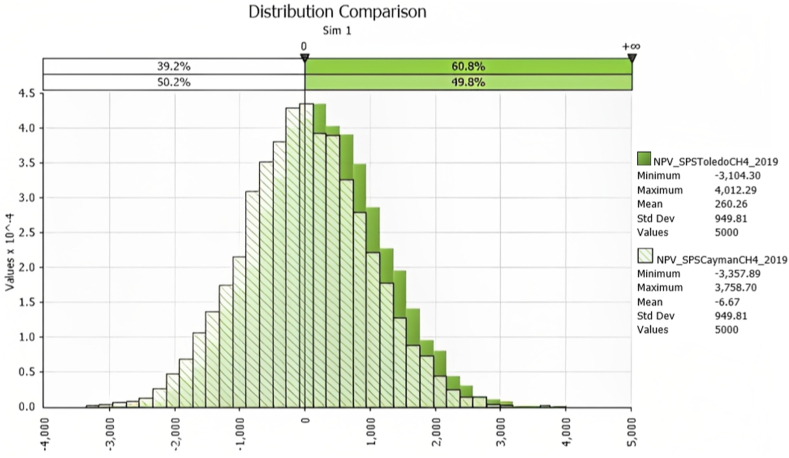
Probability density distributions for NPV for the SPS treatments under the CH_4_ reduction scenario (dark green: SPS Toledo; light green: SPS Cayman).

As the data for Scenario 1 shows, when the environmental benefit of CH_4_ reduction is included in the economic evaluation, all economic indicators improve for the SPS treatments. Particularly, the NPV for SPS Toledo increases by 741% compared to the SPS Toledo without CH_4_ reduction, and for SPS Cayman, it turns positive for the first time. Likewise, for SPS Toledo, the IRR increases by 517% and further improves for SPS Cayman. The B/C, however, only slightly increases when including CH_4_ reduction. Regarding the risk of obtaining economic loss (NPV<0), further improvements can be observed for both SPS when CH_4_ reduction is considered (Fig. 3). Nevertheless, the risk of obtaining economic loss remains high for both SPS Toledo (39.2%) and SPS Cayman (50.2%).

## 5 Discussion

This study evaluated the viability of integrating the tree legume species *Leucaena leucocephala* in two different grass monoculture cattle feeding systems, namely *Urochloa brizantha* cv. Toledo and *Urochloa* hybrid cv. Cayman, under two SPS arrangements. Particularly, financial viability of this endeavor was evaluated in two scenarios, namely (i) a base scenario, which only considered the productive parameters, and (ii) a scenario which considered productive parameters and the environmental benefit of CH_4_ emissions reductions in the SPS.

The findings of the study reveal that the introduction of SPS in the base scenario yields noticeable enhancements in profitability indicators, such as NPV, IRR, B/C, and NPV <0, regardless of the chosen grass technology. However, despite these improvements, only the SPS Toledo arrangement displays a positive NPV and IRR. It is worth noting that even this most favorable option does not exhibit strong attractiveness due to the relatively modest values of these indicators. These results exhibit some alignment with existing literature, as many studies exploring the economic viability of SPS similarly report significant enhancements, akin to the current study. Nonetheless, in most cases, these enhancements typically translate into highly positive economic indicators and overall viability [57,[96], [97], [98], [99],^95^]. The unfavorable indicators observed for SPS Cayman can be primarily attributed to its comparatively higher establishment costs in contrast to SPS Toledo. Both cases, however, may also be influenced by relatively low daily liveweight gains observed in the trial that underpins this evaluation. These observed gains average less than 240 g in both SPS, which contrasts with similar SPS setups where gains typically range between 600 and 700 g [57]. These differences could contribute to the relatively discouraging results observed in this particular study.

This rather unfavorable investment panorama, however, further changes when the monetary value of the environmental benefit of CH_4_ emissions reductions is integrated into the profitability analysis. By integrating the value of CH_4_ emissions reductions, which was estimated at > US$6, >US$24, and >US$28 per cattle, hectare, and year, respectively, the NPV of SPS Toledo increases by >700% and the one of SPS Cayman turns positive. Likewise, the other indicators further improve for both SPS. Nevertheless, this benefit alone still is not enough to make both SPS an attractive investment alternative as the relatively low IRR shows. The estimated savings in CO_2eq._ emissions of 9% in the SPS, however, are attractive when it comes to climate change mitigation. Particularly, a SPS Toledo or SPS Cayman system of 1,000 animals (which would translate into an area of 250 ha considering the stocking rate of four animals per hectare) can save 145 tons of CO_2eq._ compared with the grass monocultures, which are rather common in the Colombian cattle landscape [55,153]. This coincides with the study of Cuevas-Reyes et al. [94] who found that the profitability indicators of a SPS with *Leucaena leucocephala* and *Cynodon dactylon* significantly improve when their carbon capture potential is considered and monetized. Likewise, it coincides with Bussoni et al. [93], whose findings suggest that the reduction of CO_2eq._ emissions can be combined with the productive goals of cattle farming under SPS.

The ecosystem service of microclimatic regulation has been estimated to hold an economic value of $2,026 per hectare per year. It is important to note, however, that this value is not factored into the financial analysis. The internalization of the shade effect from trees serves as an intermediate component that does not impact the discounted NPV of SPS. This shade effect is utilized for the cattle's own intermediate consumption within the SPS. The establishment of the evaluated SPS, which yields approximately 60% shade coverage, brings about notable benefits. These advantages contribute significantly to various aspects, including (i) animal welfare improvement: SPS mitigate heat stress and reduce the presence of hematophagous flies, enhancing the well-being of the cattle [[154], [155], [156], [157], [158]], (ii) environmental enhancement: The shade cover of SPS reduces water consumption by the animals and promotes biodiversity [[159], [160], [161]], and (iii) productivity and quality enhancement: SPS positively influence productivity and quality indicators [154,159,162]. This multi-faceted impact highlights SPS's potential to contribute to climate change mitigation by curbing CH_4_ emissions [[110], [111], [112], [113]]. Additionally, SPS aid in climate adaptation by providing thermal comfort to animals amidst rising temperatures and bolstering feed availability during critical periods [158,159,163,164]. While not directly incorporated into the financial analysis, these ecosystem services reinforce the overall value and resilience of SPS systems.

To fully unlock the comprehensive environmental, economic, and productive benefits of SPS, a conducive environment for adoption must be fostered. This involves tailoring strategies to account for the distinct characteristics and disparities among adopting farmers in various regions, as well as acknowledging their unique needs, experiences, and local expertise. However, creating such an adoption-friendly environment proves to be a formidable challenge, particularly in countries like Colombia where SPS adoption rates remain generally low [39,57,65,66]. The adoption process itself encounters numerous barriers that necessitate diligent efforts to overcome, as evidenced by research findings. These barriers encompass a spectrum of challenges, ranging from financial hurdles (lack of access to credit, extended payback periods) to knowledge gaps (limited access to information, technical guidance, and extension services; intricacies of SPS practices) [65,66,73,[165], [166], [167]]. Socio-cultural factors also play a role, as traditional norms often associate pastures with tree-free landscapes [65]. Labor considerations add another layer of complexity, with SPS demanding higher labor input and facing competition from other (sometimes illegal) sectors [168]. Land tenure issues can further hinder adoption [72,73], while environmental concerns (perceived or actual) and the adaptation of species to specific environments pose additional challenges [169]. Market dynamics, legislative constraints, and a general farmer aversion to risk contribute to the array of barriers [39,65]. Addressing these multifaceted barriers requires a holistic and nuanced approach that combines targeted interventions, robust policies, and comprehensive support mechanisms. By strategically navigating these challenges, countries can effectively elevate SPS adoption rates, harness the array of benefits they offer, and ultimately establish a more sustainable and resilient agricultural landscape.

To promote the widespread adoption of SPS, a variety of instruments and incentives have been proposed and implemented through research and policy initiatives. Among these, Payments for Ecosystem Services (PES) have emerged as a notable strategy, offering strong incentives for adoption, particularly when farmers are actively engaged in their design [53,165,168,169]. Additional incentives encompass credits aimed at establishing SPS, tax benefits, commercial incentives such as price premiums, development of effective extension systems and technical support, removal of regulatory hurdles, and subsidies for trees, seeds, inputs, and equipment [39,165,170]. Research has also highlighted the importance of bolstering social networks and social capital, indicating their significant role in driving SPS adoption [66,72]. In instances where rural extension services are limited, complementary support from research initiatives can play a pivotal role in boosting adoption rates [166]. Over the past decade, Colombia has made considerable strides in scaling up SPS adoption, particularly by generating incentives to foster widespread implementation. Notably, the Sustainable Colombian Cattle Project (2010–2019) has facilitated the adoption of approximately 5,000 ha of SPS across the country [42]. The establishment of the Sustainable Cattle Roundtable in 2014 further supports the development of public policies and capacity-building efforts in sustainable cattle farming, with a particular emphasis on SPS [43,171]. This collective effort has culminated in the release of Colombia's first national-level public policy on sustainable cattle in 2022 [172]. Other significant policy advancements include the establishment of Zero-Deforestation Agreements for Beef and Dairy in 2018 [173], the development of Nationally Appropriate Mitigation Actions for cattle since 2014 [174], and the enactment of the SNIA law reforming the national agricultural innovation system in 2017 [175]. In terms of financing, a groundbreaking credit line dedicated to SPS was introduced in 2020, aimed at supporting the acquisition of planting materials [176]. Furthermore, the private sector is actively contributing to incentives, such as product differentiation and price premiums (e.g., Sustainable Cattle Initiative GANSO, AngusAzul's sustainability program), and offering technical assistance for SPS establishment (e.g., GANSO, Alquería's Heirs of Tradition program) [40,42,177,19].

While the benefits of scaling SPS are substantial, it's crucial to also acknowledge the potential unintended consequences of widespread adoption. Parodi et al. [178], for instance, advocate for implementing SPS primarily in areas unfit for crop production to mitigate unwanted competition with other agricultural systems. However, this approach may introduce negative outcomes, as evidenced by increasing deforestation trends observed when cattle intensification takes place on marginal lands [179,180], particularly when land tenure is unclear [181]. Castro-Nuñez et al. [8] highlight another potential concern, noting that the establishment of SPS can have adverse effects on forest cover in Colombia. This is attributed to improved cattle birth rates within SPS, which in turn lead to surplus calves often being sold to unsustainable fattening farms situated at the deforestation frontier. Notably, cattle farming expansion ranks among the primary drivers of deforestation in Colombia and Latin America [[2], [3], [4], [5], [6], [7], [8], [9], [10], [11], [12]]. Moreover, the productivity gains yielded by SPS could inadvertently encourage cattle farmers to further expand their operations at the expense of forests and other vital ecosystems [68], a phenomenon referred to as the Jevons paradox [182,183]. To counteract such negative and undesired consequences and to uphold the principles of sustainability in cattle farming via SPS, a strategic combination of the aforementioned incentives and robust monitoring and control mechanisms becomes imperative [68,[184], [185], [186]]. These mechanisms may include deforestation monitoring, traceability systems, and taxes targeting conventional pasture usage, among others. By implementing such measures, the potential adverse effects of SPS expansion can be mitigated, ensuring that the promise of sustainability in cattle farming is upheld.

## 6 Conclusions and recommendations

Silvo-pastoral systems are considered a sustainable production alternative for cattle systems in the global tropics and especially in Latin America. However, little is known yet about the financial viability of establishing such systems in different contexts and the added economic value of the environmental benefits and ecosystem services they provide. The present study aimed at contributing to closing this research gap by providing an economic-environmental analysis of two SPS for the Colombian context, considering the environmental benefit of methane emissions reductions and the ecosystem service of microclimatic regulation.

The study shows that when the analyzed traditional grass monocultures are transformed into the proposed SPS, the profitability indicators get significantly better, yet no outstanding financial viability was observed in this case. This, however, changes once the monetary value of CH_4_ emissions reductions is considered in the financial analysis, converting the proposed SPS into more attractive investment options. A widespread adoption of these SPS can create important socio-environmental benefits, such as the reduction of 145 tons of CO_2eq._ for every 1,000 cattle fed in these systems, valued at US$6,121 – roughly what 32 typical passenger vehicles emit per year [187]. Likewise, the suggested tree cover in the SPS generates 60% tree shade, which, per 1,000 ha creates a value of US$>2million and adds to the numerous other ecosystem services SPS provide.

However, for the scaling of SPS, numerous adoption barriers must be overcome, for which in Colombia, several public and private initiatives and programs were already established. In this regard, it is important that these endeavors consider the particularities and differences among the adopting farmers in different regions, as well as their needs, experiences, and local knowledge. It is also essential to integrate the monetary values of the environmental benefits and ecosystem services into the financial analysis of SPS, so that the stakeholders involved in the adoption process can make more informed decisions. This is, however, not always possible since technical data is not available for all environmental benefits and ecosystem services SPS offer for which it is recommended to include this in future research. To avoid unintended negative consequences in the adoption of SPS, such as deforestation, incentives (e.g., PES, credits, price premiums) need to be coupled with monitoring and control mechanisms (e.g., traceability, zero-deforestation). Only then, the sustainability claims of SPS can be upheld. Likewise, the knowledge component of SPS is very complex, and much of the adoption depends on the available knowledge, extension, and technical assistance, as well as farmer networks. A strengthening of these elements and better coordination among the actors involved is thus recommended.

## Author contribution statement

Danny Fernando Sandoval: Conceived and designed the analysis; Analyzed and interpreted the data; Contributed analysis tools or data; Wrote the paper.

Jesús Fernando Florez: Conceived and designed the analysis; Analyzed and interpreted the data; Contributed analysis tools or data; Wrote the paper.

Karen Johanna Enciso Valencia: Conceived and designed the analysis; Contributed analysis tools or data.

Mauricio Efren Sotelo Cabrera: Analyzed and interpreted the data; Contributed analysis tools or data.

Stefan Burkart: Conceived and designed the analysis; Analyzed and interpreted the data; Contributed analysis tools or data; Wrote the paper.

## Funding

This work was funded by the One CGIAR Initiatives Livestock and Climate (L&C) and Sustainable Animal Productivity (SAP). The funders had no role in the design of the study; in the collection, analyses, or interpretation of data; in the writing of the manuscript, or in the decision to publish the results.

## Data availability statement

Data will be made available on request.

## Declaration of competing interest

The authors declare that they have no known competing financial interests or personal relationships that could have appeared to influence the work reported in this paper.
